# Correlation between worsening pneumonitis and right ventricular systolic function in critically ill patients with COVID-19

**DOI:** 10.1186/s44156-024-00054-z

**Published:** 2024-08-01

**Authors:** Hazem Lashin, Jonathan Aron, Shaun Lee, Nick Fletcher

**Affiliations:** 1 Adult Critical Care Unit, St Bartholomew’s Hospital, West Smithfield, London, EC1A 7BE UK; 2 William Harvey Research Institute, Queen Mary University of London, London, UK; 3 Adult Critical Care Unit, St George’s University Hospital, London, UK; 4 Adult Critical Care Unit, Cleveland Clinic, London, UK

**Keywords:** COVID-19, Right ventricle, Echocardiography, ARDS

## Abstract

**Background:**

The pneumonitis associated with coronavirus disease 2019 (COVID-19) infection impacts the right ventricle (RV). However, the association between the disease severity and right ventricular systolic function needs elucidation.

**Method:**

We conducted a retrospective study of 108 patients admitted to critical care with COVID-19 pneumonitis to examine the association between tricuspid annular plane systolic excursion (TAPSE) by transthoracic echocardiography as a surrogate for RV systolic function with PaO_2_/FiO_2_ ratio as a marker of disease severity and other respiratory parameters.

**Results:**

The median age was 59 years [51, 66], 33 (31%) were female, and 63 (58%) were mechanically ventilated. Echocardiography was performed at a median of 3 days [2, 12] following admission to critical care. The PaO_2_/FiO_2_ and TAPSE medians were 20.5 [14.4, 32.0] and 21 mm [18, 24]. There was a statistically significant, albeit weak, association between the increase in TAPSE and the worsening of the PaO_2_/FiO_2_ ratio (r^2^ = 0.041, *p* = 0.04). This association was more pronounced in the mechanically ventilated (r^2^ = 0.09, *p* = 0.02). TAPSE did not correlate significantly with FiO_2_, PaO_2_, PaCO_2_, pH, respiratory rate, or mechanical ventilation. Patients with a TAPSE ≥ 17 mm had a considerably worse PaO_2_/FiO_2_ ratio than a TAPSE < 17 mm (18.6 vs. 32.1, *p* = 0.005). The PaO_2_/FiO_2_ ratio predicted TAPSE (OR = 0.94, *p* = 0.004) with good area under the curve (0.72, *p* = 0.006). Moreover, a PaO_2_/FiO_2_ ratio < 26.7 (moderate pneumonitis) predicted TAPSE > 17 mm with reasonable sensitivity (67%) and specificity (68%).

**Conclusion:**

In patients admitted to critical care with COVID-19 pneumonitis, TAPSE increased as the disease severity worsened early in the course of the disease, especially in the mechanically ventilated. A TAPSE within the normal range is not necessarily reassuring in early COVID-19 pneumonitis.

**Supplementary Information:**

The online version contains supplementary material available at 10.1186/s44156-024-00054-z.

## Introduction

Coronavirus disease 2019 (COVID-19), caused by the severe acute respiratory syndrome coronavirus 2 (SARS-CoV-2), often leads to pneumonitis and significantly increased admissions to hospitals and critical care units worldwide [1]. The increase in critical care admissions is attributed to respiratory compromise that requires support, ranging from additional oxygen to invasive mechanical ventilation and extracorporeal membrane oxygenation (ECMO). Also, COVID-19 often leads to dysfunction in other organs, particularly the heart and kidneys. In cases where multiple organs are affected, the risk of mortality due to COVID-19 is higher [2]. 

COVID-19 adversely affects the heart in two ways [3, 4]. Firstly, it can directly affect the myocardium. Second, it can lead to complications arising from severe lung disease, which often result in right ventricular (RV) dysfunction. These adverse effects may lead to potentially life-threatening hemodynamic instability and cardiogenic shock. In grave cases, mechanical circulatory support is necessary to sustain life. Ensuring close observation and timely intervention for patients with RV dysfunction is paramount as they are at a significantly elevated mortality risk [3–6]. 

Several factors may lead to RV dysfunction in COVID-19 patients. Hypoxia caused by pneumonitis can directly affect the RV and reduce its systolic function [7]. However, this group’s main reason for dysfunction is the rise in RV afterload [3]. Severe inflammation induced by SARS-COV-2 can lead to pulmonary vascular dysfunction and increased resistance. COVID-19 can also cause micro- or macro emboli in the pulmonary circulation, leading to increased pulmonary artery pressure. In mechanically ventilated patients, high ventilatory pressures required for adequate gas exchange can further increase pulmonary artery pressure. When combined, these factors may cause acute pulmonary hypertension and RV dysfunction.

Studies have demonstrated that COVID-19 patients in critical care may exhibit one or more echocardiographic features of RV dysfunction [4, 5]. These features include impaired RV systolic function. However, the relationship between the severity of illness and RV dysfunction has yet to be established.

We hypothesised that, as COVID-19 pneumonitis worsens, RV systolic function declines in patients admitted to critical care for respiratory support. To test our hypothesis, we conducted a retrospective single-centre cohort study that examined the association between the PaO_2_/FiO_2_ ratio as a marker of the COVID-19 pneumonitis severity and tricuspid annular plane systolic excursion (TAPSE), using transthoracic echocardiography (TTE) as a surrogate for RV systolic function. We also investigated the correlation between TAPSE and other commonly measured respiratory parameters.

## Materials and methods

### Data collection

We retrospectively reviewed consecutive patients admitted to our tertiary adult intensive care unit and diagnosed with COVID-19 pneumonitis between the 1st of February 2020 and the 28th of February 2021. Recent guidelines were used for data collection [8]. A secure web-based platform (REDCap) was used to collect and manage anonymised data [4]. The study received local research governance approval and followed the guidelines of the Declaration of Helsinki.

### Patients and clinical data

Patients diagnosed with COVID-19 pneumonitis were admitted to the critical care unit for advanced respiratory support between the 1st of February 2020 and the 28th of February 2021. Support was protocolised for spontaneously breathing patients to facemask continuous positive pressure ventilation (CPAP) of 10 cmH_2_O or 15 cmH_2_O if the body mass index (BMI) was > 35. The patients who required further support were mechanically ventilated. Demographic and clinical data that could aid in echocardiographic interpretation were collected. The collected data included age, sex, body mass index (BMI), arterial blood gases, ventilation mode and parameters, cardiovascular parameters, and outcomes. Patients were included in the study if TAPSE and PaO_2_/FiO_2_ ratios were available.

### Echocardiography

TTE studies were performed on all patients admitted to critical care with COVID-19 pneumonitis by critical care physicians trained and experienced in echocardiography and interpreted offline by accredited study team members (European Diploma in Advanced Critical Care Echocardiography [EDEC], British Society of Echocardiography level 2 or equivalent). All reviewers were blinded to the clinical parameters of the study. Data on LV ejection fraction (LVEF), LV size by visual estimation, tricuspid annular plane systolic excursion (TAPSE), RV size by visual estimation, and tricuspid regurgitation maximum velocity (TR Vmax) were collected. TAPSE was measured by M-Mode in an RV-focused view. A chamber was considered significantly dilated if classified as moderately or more dilated.

#### Endpoints

The primary endpoint of this study was the association between RV systolic function indicated by TASPE and the PaO_2_/FiO_2_ ratio as a marker for COVID-19 pneumonitis disease severity. The secondary endpoint was the association between TAPSE and other respiratory parameters.

### Statistical analysis

Continuous parameters were presented as median and interquartile range [IQR] and compared using the Mann-Whitney U test. Categorical data are presented as numbers, and percentages were compared using the chi-squared test. We tested correlations between continuous parameters using simple linear regression and correlations between continuous and categorical parameters using simple logistic regression. Linear regression analysis results are presented as r^2^, whereas those of the logistic regression analysis are presented as odds ratios (OR) and 95% confidence intervals (CI). The receiver operator curve (ROC) was used to investigate the efficacy of regression, and the results were presented as an area under the curve (AUC). In addition, ROC analysis allowed the identification of cutoffs with the best sensitivity and specificity for regression. A statistical analysis plan was defined before the analysis, and missing data were not imputed. Statistical analyses were performed using GraphPad Prism version 10 for the MacOS software package (GraphPad Software, San Diego, California, USA), and statistical significance was set at *p* < 0.05.

## Results

**Fig. 1:**
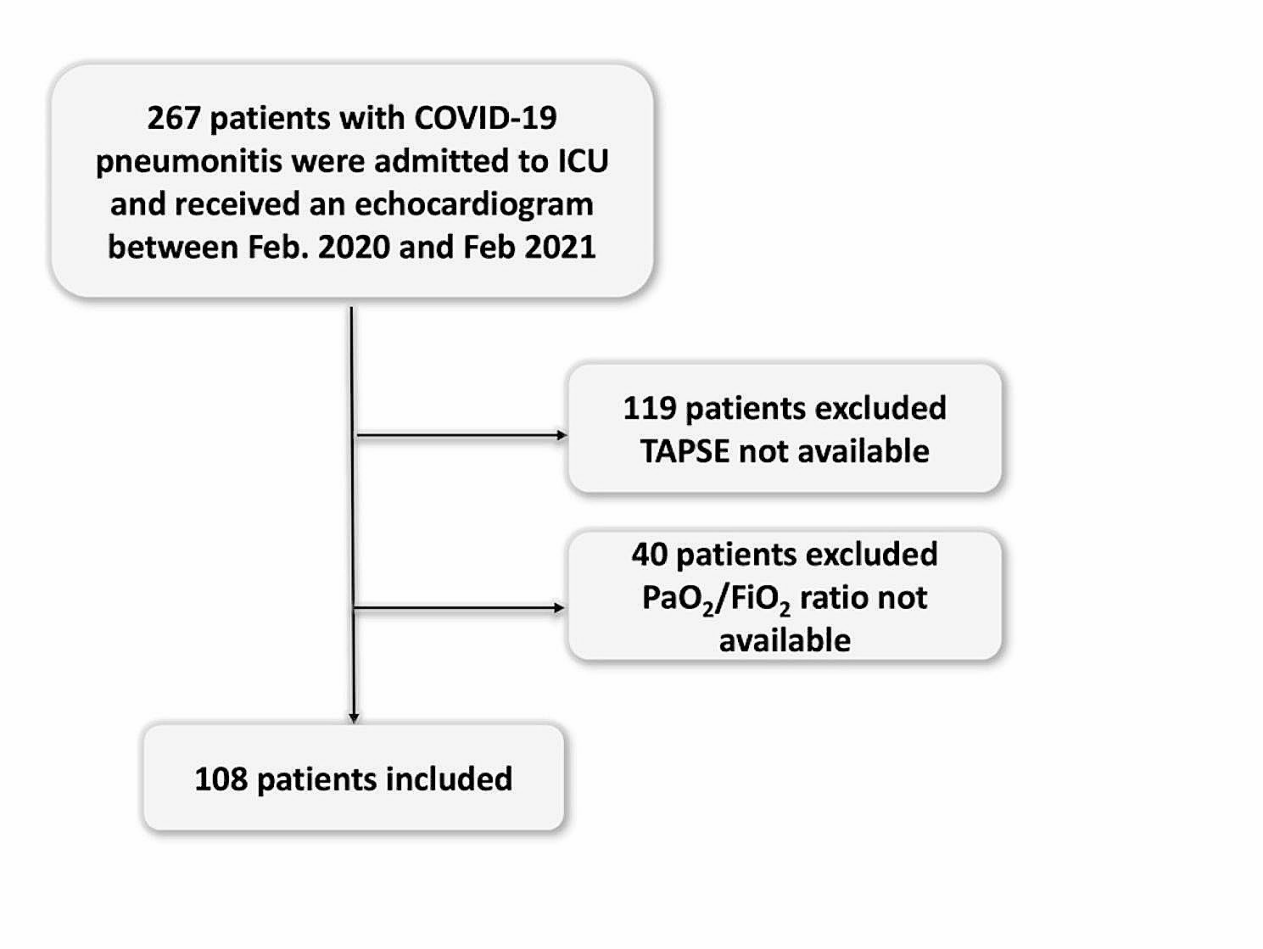
CONSORT diagram for patient inclusion and exclusion. COVID-19: coronavirus disease 2019, ICU: intensive care unit, TAPSE: tricuspid plane systolic excursion

Two-hundred and sixty-seven consecutive patients were admitted to our critical care unit with a diagnosis of COVID-19 pneumonitis and underwent echocardiography. A total of 119 patients were excluded from the study due to the unavailability of TAPSE data, followed by the exclusion of an additional 40 patients due to the unavailability of the PaO_2_/FiO_2_ ratio. Thus, 108 patients were included in the analysis (Fig. 1).

The median age was 59 years [51, 66], 33 (32%) were female, and 63 (58%) were mechanically ventilated at the time of echocardiography (Table 1). The median PaO_2_/FiO_2_ ratio was in the moderate range (20.5 [14.4, 32.0]) for acute respiratory distress syndrome (ARDS). Echocardiography was performed at a median of 3 days [2, 12] following admission to critical care. Echocardiograms revealed LVEF and TAPSE medians within the normal ranges (55% [55, 62] and 21 mm [18, 24], respectively). The LV and RV were significantly dilated (moderate or more) in 3.6% and 34% of patients, respectively. The median TR Vmax was just above the cutoff for pulmonary hypertension (2.91 m/s [2.2, 3.1]) (Table 1).

**Table 1:** Demographic, clinical, and echocardiographic parameters. IQR: interquartile range. LV: Left ventricle. LVEF: Left ventricular ejection fraction. RV: Right ventricle. TAPSE: Tricuspid annular plane systolic excursion. TR Vmax: Maximum tricuspid regurgitation velocity

Parameter	Result
Age, years [IQR]	59 [51, 66]
Female sex, n (%)	33 (31%)
Body mass index, [IQR]	27.3 [23.6, 31.6]
Mechanically ventilated, n (%) • Positive end-expiratory pressure, cmH_2_O [IQR] • Tidal volume, ml [IQR]	63 (58%) • 8 [7, 10] • 460 [400, 516]
Mortality, n (%)	49 (45%)
FiO_2_, [IQR]	0.50 [35, 63]
PaO_2_, kPa [IQR]	9.8 [8.4, 11.9]
PaCO_2_, kPa [IQR]	6.0 [4.7, 7.4]
pH, [IQR]	7.39 [7.31, 7.45]
Base excess, mmol/l [IQR]	0.8 [-2.9, 6.2]
Respiratory rate, [IQR]	24 [20, 30]
PaO_2_/FiO_2_ ratio, [IQR]	20.50 [14.44, 32.04]
Mean arterial pressure, mmHg [IQR]	77 [67, 90]
Norepinephrine support, n (%)	37 (34%)
Norepinephrine dose, mcg/kg/min [IQR]	0.13 [0.06, 0.31]
Heart rate, bpm [IQR]	90 [77, 105]
LVEF, % [IQR]	55% [55, 62]
LV significantly dilated, n (%)	4 (3.6%)
TAPSE, mm [IQR]	21 [18, 24]
RV significantly dilated, n (%)	37 (34%)
TR Vmax, m/s [IQR]	2.9 [2.2, 3.1]

**Fig. 2:**
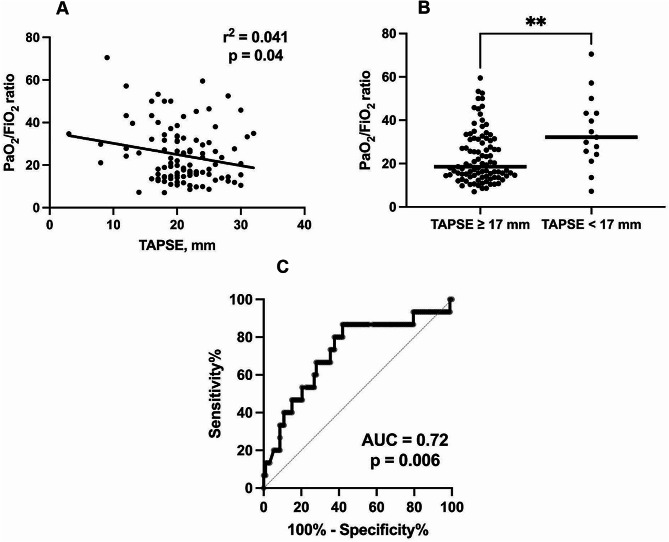
Correlation between the PaO_2_/FiO_2_ ratio and TAPSE in patients admitted to critical care with COVID-19 pneumonitis. A: Correlation between TAPSE and PaO_2_/FiO_2_. B: Comparison of the PaO2/FiO2 ratio medians between patients categorised based on TAPSE of 17 mm. C: ROC of the PaO_2_/FiO_2_ ratio prediction of TAPSE equal to or above or below 17 mm. AUC: area under the curve. ROC: receiver operator curve. TAPSE: Tricuspid annular plane systolic excursion. **: *p* < 0.01

We investigated whether the PaO_2_/FiO_2_ ratio, a marker of pneumonitis severity, correlated with TAPSE as an indicator of RV systolic function, the study’s primary endpoint. The PaO_2_/FiO_2_ ratio was significantly and negatively correlated, albeit weakly, with TAPSE in this cohort (r^2^ = 0.041, *p* = 0.04); (TAPSE increased as PaO_2_/FiO_2_ worsened) (Table 2; Fig. 2A). There were no statistically significant correlations between PaO_2_, FiO_2_, PaCO_2_, pH, respiratory rate, mechanical ventilation, and TAPSE in this cohort (Table 2), the study’s secondary endpoint.

**Table 2:** Correlation between respiratory parameters and tricuspid annular plane systolic excursion (TAPSE) in patients admitted to critical care with COVID-19 pneumonitis. CI: confidence interval

Parameter	*r*^2^ or Odds Ratio (CI)	*p* value
PaO_2_/FiO_2_	0.041	0.04
PaO_2_	0.014	0.22
FiO_2_	0.020	0.14
PaCO_2_	0.009	0.31
pH	0.005	0.46
Respiratory rate	0.024	0.35
Mechanical ventilation	1.01 (0.93, 10.9)	0.85

To further explore the association between the PaO_2_/FiO_2_ ratio and TAPSE, we categorised the patients based on a TAPSE of 17 mm (lower limit of normal). Patients with TAPSE ≥ 17 mm (*n* = 93) had a significantly lower (worse) PaO_2_/FiO_2_ ratio compared to those (*n* = 15) with TAPSE below 17 mm (18.6 [14.2, 31.2] vs. 32.1 [24.2, 43.2], *p* = 0.005) (Fig. 2B). Furthermore, univariate logistic regression revealed that the PaO_2_/FiO_2_ ratio could predict TAPSE equal to or above or below the lowest normal cutoff (17 mm), with an OR (CI) of 0.94 (0.91, 0.98) and a p-value of 0.004. ROC analysis of this model demonstrated that the PaO_2_/FiO_2_ ratio had a good AUC (0.72, *p* = 0.006) for predicting TAPSE (Fig. 2C). Moreover, a PaO_2_/FiO_2_ ratio of 26.7 could predict TAPSE equal to or above or below 17 mm with reasonable sensitivity (67%) and specificity (68%).

To further explore the correlation between TAPSE and PaO_2_/FiO_2_, we performed a subgroup analysis of two distinct patient populations: those supported by mechanical ventilation (*n* = 63) and those supported by CPAP (*n* = 45). The analysis revealed that in patients supported by mechanical ventilation, TAPSE was significantly correlated with the PaO_2_/FiO_2_ ratio (r^2^ = 0.09, *p* = 0.02). In contrast, in patients supported by facemask CPAP, the correlation was not statistically significant (r^2^ = 0.01, *p* = 0.46).

## Discussion

We conducted a retrospective study in a single centre to investigate the association between RV systolic function, indicated by the TAPSE, and the PaO_2_/FiO_2_ ratio as a marker of disease severity in patients with COVID-19 pneumonitis who required respiratory support in critical care. Initially, we hypothesised that TAPSE would decrease as the PaO_2_/FiO_2_ ratio worsened. However, our observations showed a weak but statistically significant association where TAPSE increased as the disease severity worsened early in the disease course, particularly in mechanically ventilated patients. This association was observed at a median of 3 days following admission to critical care. We observed no significant correlation between TAPSE and other respiratory parameters. Furthermore, patients with TAPSE levels equal to or above the lower limit of normal (17 mm) had significantly lower PaO_2_/FiO_2_ ratios (worse) than those above this cutoff. We also discovered that the PaO_2_/FiO_2_ ratio could predict whether TAPSE levels were equal to or above, or below the cutoff, with a good AUC of 0.72. Finally, we observed that the PaO_2_/FiO_2_ ratio at the higher end of moderate ARDS (26.7) had reasonable sensitivity and specificity for predicting TAPSE.

TAPSE is a commonly used echocardiographic surrogate of RV systolic function. This biomarker represents tricuspid annulus longitudinal displacement towards the apex and is easily measured by M-mode echocardiography at the bedside. This cohort’s median TAPSE (21 mm) lies above the lower limit of the normal range (17 mm) and within the previously reported ranges in COVID-19 patients [9–11]. In a meta-analysis of COVID-19 patients, TAPSE was independently associated with mortality [9, 12]. Where non-survivors exhibited lower TAPSE than survivors, each 1 mm decrease in TAPSE was associated with a further increase in mortality. In addition, COVID-19 patients with a lower TAPSE (19 mm vs. 21 mm) exhibited a significantly higher incidence of subsequent respiratory deterioration [13]. Furthermore, one study demonstrated a correlation between COVID-19 severity diagnosed using computed tomography (CT) and RV systolic function, in which a worse CT score was associated with lower TAPSE [14]. Based on these studies, we hypothesised that TAPSE deteriorates as the disease severity worsens. However, in the current cohort, TAPSE was negatively associated with the PaO_2_/FiO_2_ ratio, where TAPSE increased as PaO_2_/FiO_2_ worsened. Including all COVID-19 patients in critical care, rather than just those with hemodynamic instability who underwent echocardiography, as in previous studies, may explain the discrepancy between the findings of the current research and those of previous studies.

In the current study, earlier in the disease course, at a median of 3 days following admission to critical care, TAPSE increased against worsening COVID-19 pneumonitis. In a previous study, echocardiography was conducted within 24 h of admission to critical care for patients with COVID-19 pneumonitis [15]. The TAPSE values in that study were comparable to those found in the current study, and no significant differences were observed between survivors and non-survivors. Later, in the course of non-COVID-19 ARDS (within two weeks of onset), a study demonstrated that TAPSE correlated positively with the PaO_2_/FiO_2_ ratio [16]. This was reflected in another cohort of COVID-19 patients; TTEs were conducted at a later stage of the illness, at a mean of six days after critical care admission, to assess hemodynamic compromise [17]. As the PaO_2_/FiO_2_ ratio deteriorated, the fractional area change (FAC), another marker of RV systolic function, also worsened. Moreover, in another small study, all patients with COVID-19 pneumonitis underwent serial echocardiography in the intensive care unit [18]. This study’s mean initial TAPSE was comparable to the current study and was similar between survivors and non-survivors (22 mm vs. 21 mm, respectively). However, in the final scan, the TAPSE of survivors was maintained at 23 mm, while that of non-survivors dropped to 19 mm (a statistically significant drop). These results indicate that RV contractility initially increases in the face of worsening ARDS to enhance pulmonary blood flow, improve gas exchange, and overcome the pulmonary vascular dysfunction associated with ARDS [19]. However, the RV is exhausted in some patients, leading to a lower TAPSE and higher mortality risk in COVID-19. Therefore, when interpreting TAPSE, it is essential to consider the timing of echocardiography in relation to ARDS. A high TAPSE reading early in the disease course may indicate a high RV effort or a hyperdynamic RV, leading to later exhaustion and hemodynamic compromise in some patients.

The guidelines state that a TAPSE measurement of less than 17 mm indicates impaired RV systolic function [20]. However, this cutoff was developed based on studies of outpatient populations who are stable and not unwell, and their cardiovascular loading conditions differ significantly from those of critical care patients. Due to critical illness, sedation, invasive ventilation, changes in preload, and the effects of vasoactive medications, our patients’ loading conditions were different from those of outpatient populations. Critical care echocardiography practice adopts this cutoff to define abnormal RV function, but it may not be relevant to critical care situations. In critical illness situations where the RV does not need to work hard, TAPSE may fall below the guidelines cutoff without indicating an impairment or abnormality. This variation is also observed in some healthy athletes whose LV ejection falls below the abnormality limit set by the guidelines for the general population while their function remains normal [21]. The guidelines’ recommended normality ranges may not always apply to critical illness. This highlights the need for further research to improve our understanding of cardiac function and its response to changes in loading conditions over time. Further exploration of increased RV function or hyperdynamic RV may be necessary in the context of critical illness and ARDS.

This study has some limitations that may affect its generalisability. This was a single-centre retrospective study. Other RV parameters were not measured, such as right ventricular tissue Doppler systolic prime velocity, right ventricular outflow tract Doppler, and RV strain by speckle tracking. Some of these limitations may be explained by the impact of the COVID-19 pandemic on ICU staff workload and their ability to collect and record data. In addition, the British Society of Echocardiography advised the adoption of focused echocardiograms during the pandemic, which may have affected echocardiographic data. Furthermore, fewer patients had TAPSE measurements below the cutoff of 17 mm, which could introduce bias. Moreover, further bias may originate from excluding patients where TAPSE was not or could not be measured.

Our study revealed a weak but statistically significant negative correlation between RV systolic function, measured by TAPSE, and the severity of COVID-19 pneumonitis, expressed as the PaO_2_/FiO_2_ ratio in the early stages of the disease. We observed that, as the PaO_2_/FiO_2_ ratio worsened, there was an increase in TAPSE, more so in the mechanically ventilated. This finding could alert clinicians to the possibility of RV exhaustion in patients with worsening ARDS, even if TAPSE is normal in the early phase of the disease. Therefore, RV protection measures should be considered before hemodynamic changes occur. This study also opens the door to researching the RV response to ARDS over time, and further research is required to determine how TAPSE, and other RV function parameters respond to ARDS as it progresses. Moreover, critical care-specific guidelines for measuring RV function require further investigation.

## Conclusion

In this retrospective study conducted in a single centre, we observed a weak but statistically significant negative association where RV systolic function indicated by TAPSE increased as the PaO_2_/FiO_2_ ratio, a surrogate for disease severity, worsened in patients admitted to critical care with COVID-19 pneumonitis, particularly in patients who required mechanical ventilation. Patients with TAPSE levels equal to or above the lower limit of normal (17 mm) had significantly worse PaO_2_/FiO_2_ ratios than those below the guidelines’ cutoff. We also discovered that the PaO_2_/FiO_2_ ratio could predict whether TAPSE levels were equal to or above, or below the cutoff (17 mm), with a good AUC of 0.72. Finally, we observed that the PaO_2_/FiO_2_ ratio at the higher end of moderate ARDS (26.7) had reasonable sensitivity and specificity for predicting TAPSE. These observations indicate that the TAPSE above the normal cutoff is not necessarily reassuring in the context of ARDS and may assist clinicians in managing the RV in this cohort. Further research is required to corroborate these observations in a prospective cohort and study RV changes in correlation with disease severity over time.

The Authors declare that there is no conflict of interest.

### Electronic supplementary material

Below is the link to the electronic supplementary material.

Supplementary Material 1

## Data Availability

Data is available from the corresponding author upon reasonable request.
