# Supplementary material for: Correlation between worsening pneumonitis and right ventricular systolic function in critically ill patients with COVID-19
Source: Echo Res Pract. 2024 Aug 1;11:19. doi: 10.1186/s44156-024-00054-z (PMC11293088; doi:10.1186/s44156-024-00054-z)
Supplement: Supplementary file 1 — Supplementary Material 1 [file 44156_2024_54_MOESM1_ESM.pptx]

## Slide 1
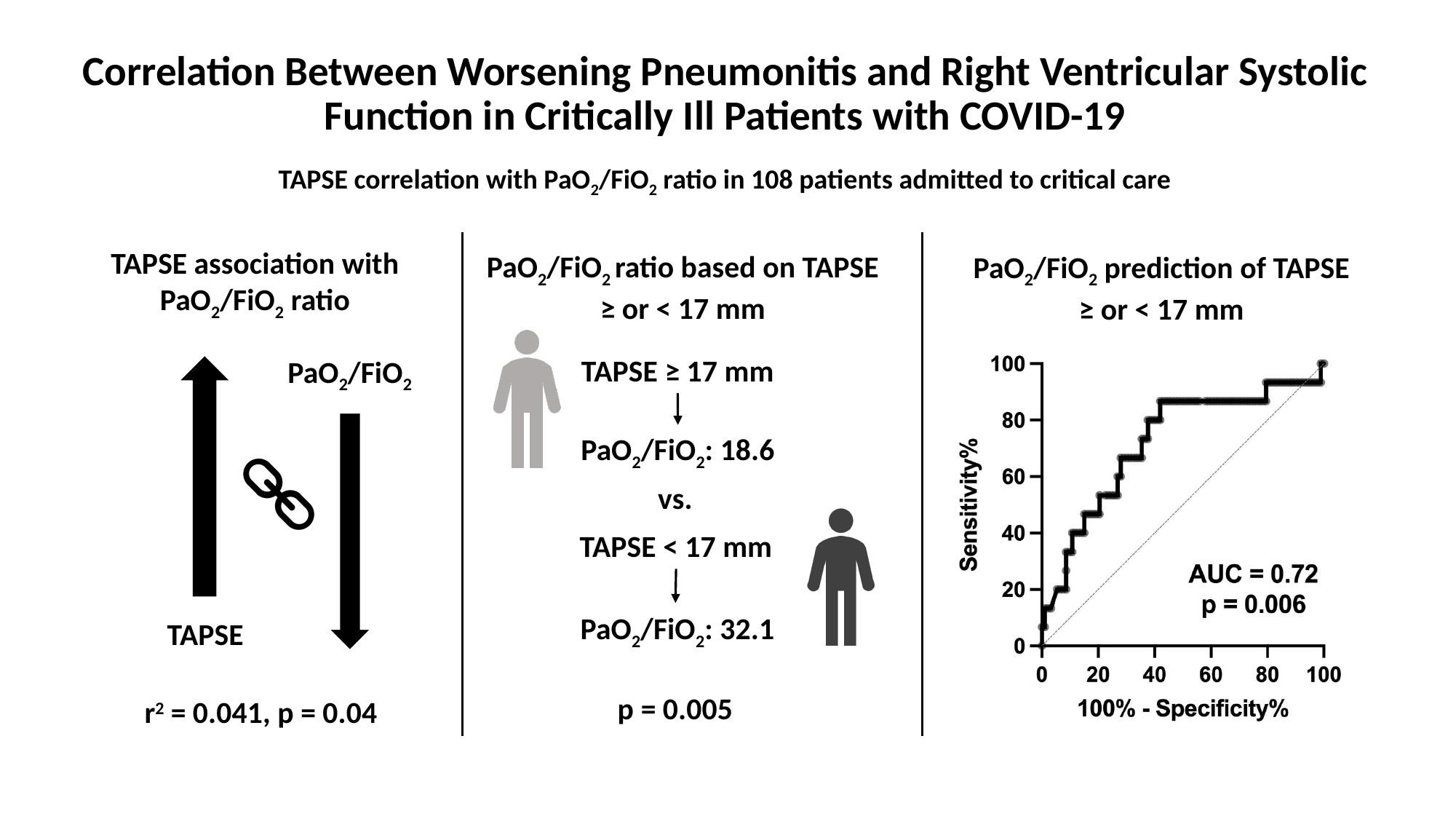

# Correlation Between Worsening Pneumonitis and Right Ventricular Systolic Function in Critically Ill Patients with COVID-19 TAPSE correlation with PaO2/FiO2 ratio in 108 patients admitted to critical care
TAPSE association with PaO2/FiO2 ratio
PaO2/FiO2 ratio based on TAPSE ≥ or < 17 mm
PaO2/FiO2 prediction of TAPSE ≥ or < 17 mm
TAPSE ≥ 17 mm
PaO2/FiO2
PaO2/FiO2: 18.6
vs.
TAPSE < 17 mm
PaO2/FiO2: 32.1
TAPSE
p = 0.005
r2 = 0.041, p = 0.04
